# Optimizing Psychopharmacotherapy in Special Populations: Educating Language Models for Tailored Treatment Approaches

**DOI:** 10.1192/j.eurpsy.2025.248

**Published:** 2025-08-26

**Authors:** G. Vannini

**Affiliations:** Swiss Timing Ltd, Corgemont, Switzerland

## Abstract

**Abstract:**

In recent years, artificial intelligence has increasingly demonstrated its potential to transform healthcare, particularly in the domain of psychopharmacotherapy. This workshop explores the integration of language models into the personalization of treatment strategies for special populations, including those with complex comorbidities or unique pharmacokinetic profiles. By educating language models with domain-specific data, we can enhance their capacity to support clinicians in decision-making processes, optimize treatment outcomes, and reduce the risk of adverse drug reactions. Attendees will gain insights into the methodologies for training AI models on tailored datasets, addressing challenges in bias mitigation and ethical considerations, and leveraging these tools for real-world clinical applications. This session aims to foster a deeper understanding of how cutting-edge AI can reshape psychopharmacological practices and empower clinicians with data-driven insights.

**Disclosure of Interest:**

None Declared

